# Is There a “Right” Side of Communicating Friendship? Lateralization of Social Interactions in Zoo *Barbary macaques* (*Macaca sylvanus*)

**DOI:** 10.3390/ani11113288

**Published:** 2021-11-17

**Authors:** Marzia Baldachini, Barbara Regaiolli, Miquel Llorente, David Riba, Caterina Spiezio

**Affiliations:** 1 Fundació UdG: Innovació i Formació, Universitat de Girona, 17003 Girona, Spain; marziabaldachini@gmail.com (M.B.); miguel.llorente@udg.edu (M.L.); 2 Research & Conservation Department, Parco Natura Viva—Garda Zoological Park, Bussolengo (VR), Loc. Figara 40, 37012 Verona, Italy; caterina.spiezio@parconaturaviva.it; 3 Serra Húnter Fellow, Facultat d’Educació i Psicologia, Universitat de Girona, 17004 Girona, Spain; 4 Institut de Recerca i Estudis en Primatologia—IPRIM, 17006 Girona, Spain; 5 Facultat de Lletres, Universitat de Girona, 17004 Girona, Spain; david.ribacano@udg.edu

**Keywords:** *Barbary macaques*, laterality, approach, proximity, affiliative contacts, social hierarchy

## Abstract

**Simple Summary:**

The aim of the current study was to investigate the side (left or right) and sagittal preference (front or rear) of adult *Barbary macaques* (*Macaca sylvanus*) during different types of social interactions. This study would improve our knowledge of the implication of emotions, through the investigation of behavioral lateralization, during social interactions and communication in macaques. No side preferences were found for any social interaction, suggesting that both hemispheres might be complemental and balance each other during intraspecific communication. For the sagittal preference, we found that macaques are kept in front rather than on the rear by close conspecifics, presumably due to the need to detect emotions and intentions of conspecifics during social interactions.

**Abstract:**

Social laterality in non-human primates has started to attract attention in recent years. The positioning of individuals during social interactions could possibly suggest the nature of a relationship and the social ranking of the subjects involved. The subjects of the present study were 12 adult *Barbary macaques* (*Macaca sylvanus*) housed in a zoological garden. We carried out fourteen 210-min video-recorded sessions and we used a focal animal sampling method to collect the position of the subjects during different social interactions. Data on the position of each macaque during three types of social interactions were collected (approach, proximity and affiliative contacts). Moreover, we focused on the outcomes of dyadic agonistic encounters to build the hierarchy of the colony. For each social interaction, two conditions were considered: the side preference (being kept on the left or on the right) and the sagittal preference (being kept in front or on the rear). Bouts of preference of different positions were collected for different social interactions (approach, proximity and contacts). No group-level side preferences were found for any social interaction, suggesting that both hemispheres might be complemental and balance each other during intraspecific communication. For the sagittal preference, we found a group-level bias for proximity, with macaques being kept in front rather than on the rear by close conspecifics. This might be due to the need to detect emotions and intentions of conspecifics. Moreover, high-ranking individuals are kept more frontally than on the rear when in proximity with other macaques. More studies are needed to better investigate social laterality, possibly distinguishing more categories of social interaction, and detecting other variables that might influence the positioning preferences.

## 1. Introduction

Hemispheric specialization is intended as the division of roles between the two cerebral hemispheres, with the left side of the brain involved for example in the process of routine actions whereas the right side deals with spatial cues of the environment and unexpected stimuli, and in some contexts has been suggested to control social stimuli [1]. Thus, choosing to use one side of the body or the other could be convenient in certain situations as it would facilitate behaviors under the control of a specific hemisphere [2,3,4].

Non-human primates living in complex social groups and showing a large repertoire of social activities can be an ideal model to study laterality of social behaviors, and its evolutionary emergence in humans. Indeed, the behavioral responses to social stimuli have been found to be lateralized in several taxa, with each hemisphere dealing with recognition of individuals (in terms of identification but also social rank), interpretation of facial expressions and management of affiliative and agonistic interactions [3,5]. A tendency to keep conspecifics in the left visual field has been reported in different vertebrate species, such as amphibians [6], reptiles [7,8,9], birds [10] and mammals [11,12]. In non-human primates, asymmetries in the display of facial expressions have been reported in different species, with the left side of the face producing facial expression earlier and more intensively than the right side in monkeys [13,14,15] and great apes [16]. Within social interactions, in wild chimpanzees the nature of social relationships influenced laterality, with a prevalence of right-handed gestures in context requiring efficient communication [17]. In the context of mother–infant interactions, different studies on non-human primates reported a left-side bias in both maternal cradling and nipple preference of the infants (for review see [18,19]). Regarding social dynamics of Old-World monkeys, gelada baboons (*Theropithecus gelada*) showed a preference for the left visual field during agonistic interactions [20]. Similarly, a study on red-capped mangabeys (*Cercocebus torquatus torquatus*) and grey-cheeked mangabeys (*Lophocebus albigena albigena*) revealed that individuals were approached more frequently from their left than from their right [21], suggesting a right-hemisphere involvement in the process of social stimuli. The same authors also investigated sagittal preference in these species, reporting that mangabeys were approached more frequently from the front than from behind [21] and the same finding was found also in chimpanzees (*Pan troglodytes*) and gorillas (*Gorilla gorilla*) [22]. As positioning in front or on the rear of conspecifics may influence individual recognition, detection of intentions and thus eye/side use, sagittal preference could be relevant and complementary in the study of social laterality. 

Non-human primate visual perception is similar to that of humans and is unique among mammals. The photoreceptor distribution and organization together with forward-facing eyes with overlapping visual fields and a high binocular convergence ensure sharp central vision in bright light conditions and color vision [23]. Moreover, binocular vision is a fundamental component of primate sight, allowing hand-eye coordination, and the perception of the self within the environment [24].

In complex social systems such as those characterizing non-human primates’ colonies, social rank can affect the emotional state of the group members and therefore their social laterality. For example, the side of approach and the sagittal preference of mangabeys varies according to the social rank, with higher ranking individuals being approached more frequently from their left and preferring to keep conspecifics behind rather than in front [21]. 

The aim of the current study was to investigate the side (left or right) and sagittal preference (front or rear) of adult *Barbary macaques* (*Macaca sylvanus*) during different types of social interactions. In particular, we investigated whether the subjects were approached or contacted by conspecifics on their left, right, in front or on the rear. Similarly, we evaluated whether macaques were kept on the left/right or in front/on the rear when in proximity of conspecifics. Based on previous literature on Old World monkeys [13,14,15,20,21], we expect that macaques would prefer to have conspecifics laterally on the left visual field, especially during approaches, and keep them on the front side when in sagittal position. Moreover, the social rank of the macaques is supposed to affect both side and sagittal preferences, with high-ranking individuals being approached mainly on the left and keeping conspecifics behind rather than in front. 

## 2. Materials and Methods

### 2.1. Subjects and Housing

The study was carried out in November and December 2017 with a colony of 12 adult *Barbary macaques*, housed at Parco Natura Viva—Garda Zoological Park (Bussolengo—VR, Italy). The colony consisted of nine females and three males of different ages (Table 1). All subjects were born in zoos and parent-reared. The macaques were housed in a 1560 m^2^ grassy sloping enclosure containing several trees and shrubs, ropes, rocks, caves and shelter areas, trunks, and a water pool. Macaques were fed twice a day with fresh fruit and vegetables and food was provided at six feeding points consisting of wire-mesh baskets. Supplemental food items were seeds, insects, boiled eggs, potatoes, and monkey pellets, nuts, and occasionally dairy products. Water was available ad libitum. Macaques were provided with different types of environmental enrichments daily, consisting of foraging enrichment (small food items scattered around in the enclosure or hidden in straw mounds) and manipulative devices containing food (e.g., paper cups and bags, cloths, cones). The zookeepers entered the enclosure only for husbandry procedures (feeding and cleaning) and the animals were not used to directly interacting with humans.

### 2.2. Data Collection

We video-recorded the observation session mounting a digital video camera (Sony Handycam FDR-AX53) on a tripod. The camera was fixed and wide angle, covering the central area (approximately 75%) of the enclosure. We recorded the colony for 14 non-consecutive days, twice a day: 10.30–12.30 and 14.00–15.30. Thus, we obtained 14 120-min morning recordings and 14 90-min afternoon recordings for a total of 210 min per daily session. We analyzed each daily session (morning + afternoon recordings) for each macaque, and we collected data from the video-recordings using a continuous focal animal sampling [25].

**Figure 1:**
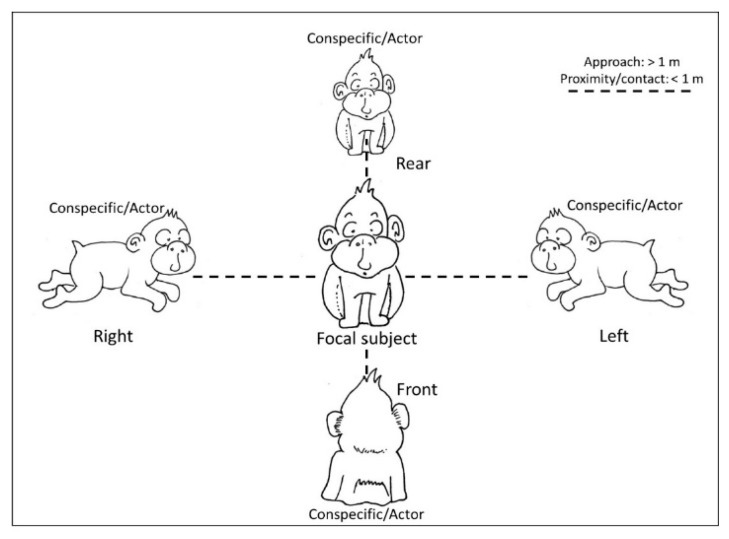
Schematic representation of the interactions considered in the current study. We recorded whether macaques (conspecific/actors) were in the left or right visual field of the focal subject or whether they were in front or on the rear of the focal subject during social interactions (approaches, proximity, and social contact such as grooming and embraces).

We collected the position of macaques with respect to conspecifics during different social interactions, particularly approach, proximity and affiliative contacts. In particular, we recorded whether macaques were in the left or right visual field of the focal subject or whether they were in front or on the rear of the focal subject during social interactions. We considered an approach when a macaque moved towards another individual; each approach ended when the approaching individual was at a distance inferior or equal to one meter. Proximity was intended as the macaque being near conspecifics, at one meter or less (Figure 1). Affiliative contacts included positive physical interactions with conspecifics that could be biased on one side of the body, specifically grooming and embraces [26,27,28]. We recorded grooming when a macaque was on one side of another individual and manipulated its fur with hands or mouth; an embrace was recorded when a macaque wrapped an arm around the body of another individual. For each interaction we recorded the recipient (focal subject), the actor and the position of the actor with respect to the recipient (Figure 1). In other words, we collected the position of the focal macaque when receiving the interaction by a conspecific and we identified two conditions: the Side Preference, in which the focal subject (recipient of an interaction) keeps a conspecific on the right or on the left and the Transversal Preference, referring to the sagittal positioning of the macaques, in which the focal subject could receive the interaction on the front or on the rear. For each social interaction, we focused only on bouts of position preference, intended as the first event of a series, as the following events could be not independent from the first choice. As in our previous study on this colony, “we collected data on dyadic agonistic encounters, focusing on behaviors that involve a clear winner (e.g., slapping, grabbing, chasing, dominance mount), with an animal directing an aggressive behavior toward another individual (the receiver/victim), which flees or moves away [28,29]”. We collected the identity of both actors and receivers and entered the outcomes of such dyadic interactions in a matrix to calculate the CBI [30,31], given by the formula CBI = (B + b + 1)/(L + l + 1). In the CBI formula, “B = number of individuals whom the subject dominates, b = number of individuals who those dominated by the subject in turn dominate, L = number of individuals who dominate the subject, l = number of individuals who dominate those dominating the subject” [30] (p. 632). The higher the CBI of an individual, the higher the rank in the social group. If the position of one of the subjects involved in any type of social interaction described above was ambiguous (e.g., it was not possible to establish whether a macaque was on the right or on the left of the focal subject) the data were excluded from the dataset and therefore from the analyses.

### 2.3. Data Analysis

To verify the presence of biases in the position of macaques, for each social interaction (Approach, Contact and Proximity) we calculated a Side Index (SI) (right-left preferences) and a Transversality Index (TI) (sagittal positioning: front-rear preference). The SI was given by the formula (R − L)/(R + L), where “R” and “L” indicating the frequency of bouts in which a subjects kept a conspecific on the right or on the left side, respectively. Similarly, the TI was given by the formula (F − Re)/(F + Re), with “F” and “Re” indicating the frequency in which a subject kept a conspecific on the front or on the rear, respectively. We used absolute values of SI and TI (ABS-SI and ABS-TI) as a measure of the strength of preference. In addition, we used binomial *z*-scores to classify the macaques as left-preferent/rear-preferent (*z* ≤ −1.96), right-preferent/front-preferent (*z* ≥ 1.96) or ambipreferent (−1.96 < *z* < 1.96) [32,33].

As Shapiro–Wilk goodness-of-fit tests revealed that data were normally distributed (SI: Approach: *p* = 0.75; Prox.: *p* = 0.73; Cont.: *p* = 0.18; ABS-SI: Approach: *p* = 0.31; Prox.: *p* = 0.11; Cont.: *p* = 0.52; TI: Approach: *p* = 0.71; Prox.: *p* = 0.43; Cont.: *p* = 0.47; ABS-TI: Approach: *p* = 0.19; Prox.: *p* = 0.09; Cont.: *p* = 0.08), we performed the group-level analysis using parametric statistical tests. 

To assess the presence of biases in side and sagittal preference at the group level, we used the one sample t-tests on the SI and the TI [18]. To verify the consistency of side/sagittal preference among different social interactions we used the one-way ANOVA for independent samples with a Tukey HSD test serving as the post hoc test. We carried out the same analysis using the ABS-SI and ABS-TI to assess variations in the strength of laterality among different interactions. Finally, we used Pearson correlation to investigate the relationship between both the SI and TI and the CBI (social rank). All tests were two tailed and the significance level was set at *p* < 0.05.

## 3. Results

### 3.1. Side Preferences during Social Interactions

At the individual level, we found no significant side biases during approach and proximity for any of the subjects, whereas two macaques received affiliative contacts significantly more on the left side than on the right side (Table 1).

**Figure 2:**
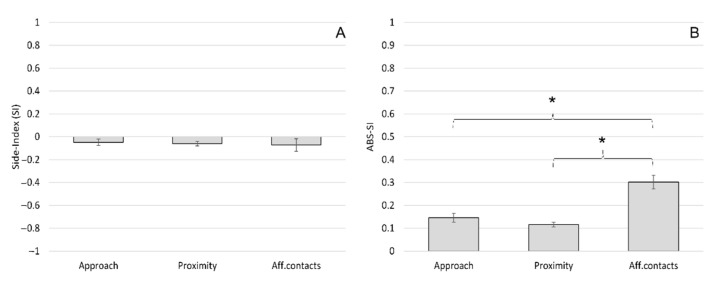
Side preference during social interactions in *Barbary macaques*. Mean Side Index (SI) (**A**) and mean ABS-SI (**B**) of different social interactions (approach, proximity and affiliative contact). Bars indicate standard error. Asterisks (*) indicate significant differences between social interactions (Tukey post hoc test: *p* < 0.05).

The mean ± SE of the SI was −0.05 ± 0.05 for approach, −0.06 ± 0.04 for proximity and −0.07 ± 0.11 for affiliative contacts (Figure 2A). The mean ± SE of the ABS-SI was 0.15 ± 0.12 for approach, 0.12 ± 0.02 for proximity and 0.30 ± 0.06 for affiliative contacts (Figure 2B). The one-sample *t*-test revealed no group-level biases in the distribution of the SI for approach (*t*(11) = −0.854; *p* = 0.411), proximity (*t*(11) = −1.516; *p* = 0.158) and affiliative contacts (*t*(11) = −0.656; *p* = 0.525). The ANOVA between different social interactions revealed no significant differences in the SI (*F*(2) = 0.03; *p* = 0.970) (Figure 2A), whereas we reported a significant difference considering the ABS-SI (*F*(2) = 5.19; *p* = 0.011); Tukey HSD post hoc tests revealed that the ABS-SI for affiliative contacts were significantly higher than the ABS-SI for approach and for proximity (Figure 2B).

### 3.2. Sagittal Preferences during Social Interactions

At the individual level, we found a significant bias in the TI for 1 out of 12 macaques (8%) that was approached significantly more on the rear than in front (Table 1). For proximity, we found that 3 out of 12 macaques (25%) had a significant bias in the TI and were kept by near conspecifics on the front rather than on the rear (Table 1). For affiliative contacts, we found that two out of nine macaques (22%) had a significant bias in the TI: one received significantly more affiliative contacts on the front, one on the rear side.

**Figure 3:**
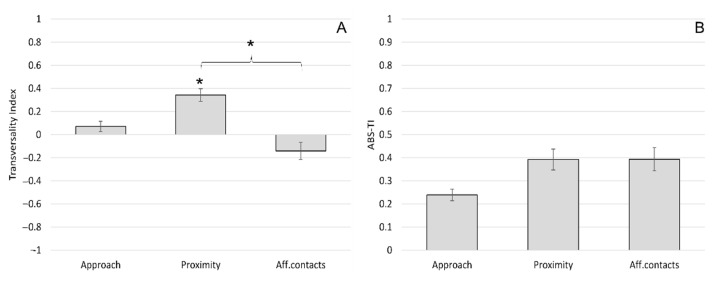
Sagittal preference during social interactions in *Barbary macaques*. Mean Transversality Index (TI) (**A**) and mean ABS-TI (**B**) of different social interactions (approach, proximity and affiliative contact). Bars indicate standard error. Asterisks (*) indicate significant group-level biases and significant differences between social interactions (Tukey post hoc test: *p* < 0.05).

The mean ± SE of the TI was 0.07 ± 0.09 for approach, 0.34 ± 0.11 for proximity and −0.14 ± 0.15 for affiliative contacts (Figure 3A). The mean ± SE of the ABS-TI was 0.24 ± 0.05 for approach, 0.39 ± 0.09 for proximity and 0.39 ± 0.10 for affiliative contacts (Figure 3B). The one-sample *t*-test revealed a significant group-level bias for proximity, with macaques being kept in front by conspecifics rather than on the rear (*t*(11) = 3.233; *p* = 0.008). We found no significant biases for approach (*t*(11) = 0.817; *p* = 0.431) and affiliative contacts (*t*(11) = −0.928; *p* = 0.373). The ANOVA between different social interactions revealed a significant differences considering the TI (*F*(2) = 4.19; *p* = 0.024). Tukey HSD post hoc tests showed that the TI for proximity was significantly higher than the TI for affiliative contacts (Figure 3A). When considering the ABS-TI, we found no significant differences between social interactions (*F*(2) = 1.09; *p* = 0.348) (Figure 3B).

### 3.3. Side/sagittal Preference and Social Rank

We investigated the relationship between side and sagittal preferences and social rank using Pearson correlations with both SI and TI and the CBI.

**Figure 4:**
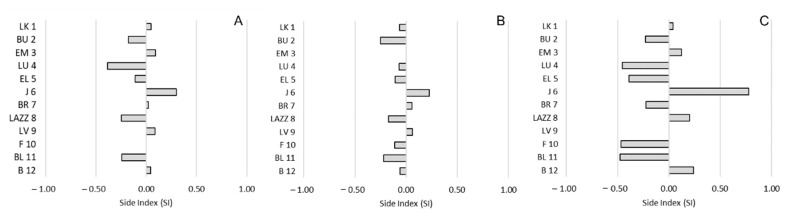
Bias of side preference (SI) and social hierarchy in *Barbary macaques*. Individuals (y-axis) are presented according to their social rank from 1 (the most dominant macaque) to 12 (the most dominated macaque), based on the CBI. (**A**): approach; (**B**): proximity; (**C**): affiliative contacts.

**Figure 5:**
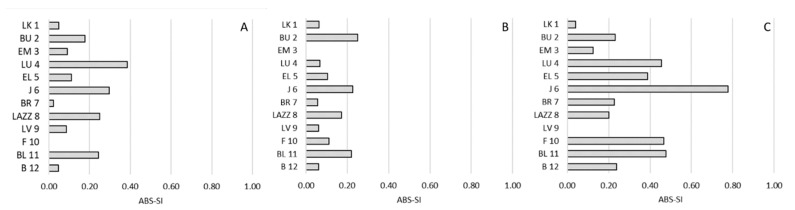
Strength of the bias of side preference (ABS-SI) and social hierarchy in *Barbary macaques*. Individuals (y-axis) are presented according to their social rank from 1 (the most dominant macaque) to 12 (the most dominated macaque), based on the CBI. (**A**): approach; (**B**): proximity; (**C**): affiliative contacts.

Regarding side preference, we found no significant correlations between the SI and the CBI for any social interaction (approach: *R*(12) = −0.011; *p* = 0.975; proximity: *R*(12) = −0.265; *p* = 0.414; affiliative contacts: *R*(12) = −0.008; *p* = 0.980;) (Figure 4). Similarly, we reported no significant correlations considering the ABS-SI (approach: *R*(12) = −0.136; *p* = 0.676; proximity: *R*(12) = 0.071; *p* = 0.827; affiliative contacts: *R*(12) = −0.396; *p* = 0.203) (Figure 5).

**Figure 6:**
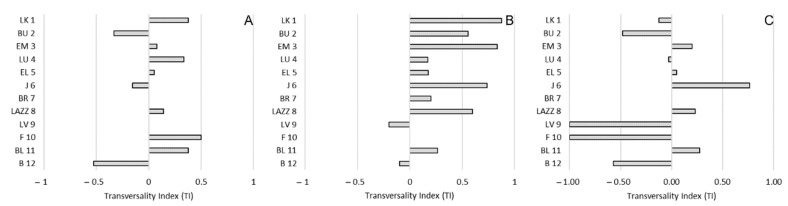
Bias of transversality preference (TI) and social hierarchy in *Barbary macaques*. Individuals (y-axis) are presented according to their social rank from 1 (the most dominant macaque) to 12 (the most dominated macaque), based on the CBI. (**A**): approach; (**B**): proximity; (**C**): affiliative contacts.

**Figure 7:**
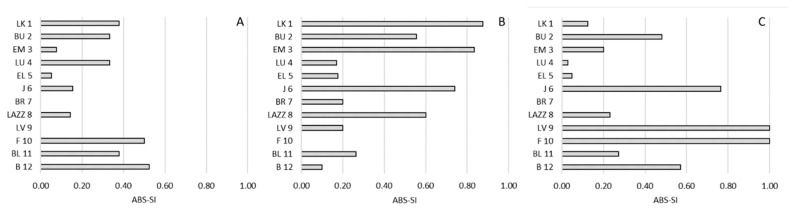
Strength of the bias of transversality preference (ABS-TI) and social hierarchy in *Barbary macaques*. Individuals (y-axis) are presented according to their social rank from 1 (the most dominant macaque) to 12 (the most dominated macaque), based on the CBI. (**A**): approach; (**B**): proximity; (**C**): affiliative contacts.

Regarding sagittal preference, we found a borderline significant positive correlation between the TI and the CBI for proximity (*R*(12) = 0.558; *p* = 0.059), whereas we reported no significant correlations for approach (*R*(12) = 0.033; *p* = 0.919) and affiliative contacts (*R*(12) = −0.073; *p* = 0.822) (Figure 6). Similarly, considering the strength of the preference, we found a borderline significant positive correlation between the ABS-TI and the CBI for proximity (*R*(12) = 0.574; *p* = 0.051), whereas we reported no significant correlations for approach (*R*(12) = 0.247; *p* = 0.439) and affiliative contacts (*R*(12) = −0.211; *p* = 0.510) (Figure 7).

## 4. Discussion

The current study investigated, for the first time, the manifestation of sagittal positioning (front/back) and lateralized positioning (left/right) associated with social stimuli in *Barbary macaques*. We analyzed frequencies of social interaction during naturalistic social encounters. Regarding side preference, we found a lack of significant side biases in social interactions in *Barbary macaques*. For the sagittal preference, we found that macaques were kept by conspecifics significantly more frequently in front than on the rear, when in proximity. In addition, we reported differences in the side and sagittal preferences among different social interactions, considering both the direction and strength of the biases.

The general lack of side biases (right/left) at both group and individual level indicates that macaques interact with conspecifics keeping them either in their left or right visual field, without any preference. This finding disagrees with previous studies on monkeys [20,21,34], and great apes [22], indicating a left-side bias during the interaction with other individuals and suggesting a right hemisphere involvement in the process of emotions deriving from social interactions [35]. The need of affiliation and the performance of dominance behaviors are both basic needs of social species such as primates and are mediated by brain structures located in the right and left hemisphere, respectively. This could lead to conflictual situations during the communication and interaction with conspecifics, with the involvement of both hemispheres determining a lack of side preference [36,37]. Despite the lack of significant biases, the strength of the side preference of macaques varied among different interactions, as the biases in affiliative contacts were more pronounced than the biases in approaches and proximity. Thus, differences between types of social behaviors and associated interaction might be leading factors in determining social laterality [34,38]. More studies are needed involving a greater number of individuals and focusing on a wider range of social interactions related to both positive and negative emotions.

For the sagittal preference, we found a group-level bias when considering proximity, with macaques preferring to have conspecifics in front when one meter or less from them. The reported group-level bias for being kept by conspecifics more frontally than behind is in line with previous research on mangabeys [21] as well as chimpanzees and gorillas [22]. Having conspecifics in front would be advantageous as it improves the identification of individuals and their facial expressions, improves communication and facilitate the prediction of possible aggressive reactions [21,22]. It has been suggested that more tolerant social species such as bonobos and Bonnet monkeys (*Macaca radiata*) tend to face each other to a greater extent than more despotic species, such as chimpanzees and pig-tailed macaques (*M. nemestrina*) [39,40]. For example, bonobos (*Pan paniscus*) have been found to prefer face-to-face grooming (grooming of the face and front side of the body) more than chimpanzees, showing more frequently a face-to-back grooming (grooming of the ano–genital area) [40]. Face-to-back grooming allows the protection of more vulnerable body sites (frontal sites) and avoid eye contact between conspecifics. Similar findings have been found in Bonnet macaques and pig-tailed macaques [39]. The fact that *Barbary macaques* are considered among the most tolerant species of macaques [41,42] can partially explain the preference for staying in front of close conspecifics, as reported for grooming in bonobos and in Bonnet macaques. Moreover, *Barbary macaques* were kept by conspecifics frontally rather than behind significantly more when in proximity than during affiliative contacts. It is possible that when near to each other macaques have a greater uncertainty on the reaction and possible development of any interaction, whereas during affiliative contacts the outcome of the interaction is yet clear. Thus, sagittal preference could be more pronounced and determinant in proximity than during other kind of interactions. 

We found no correlation between the Side Index and the CBI, suggesting that social rank seems not to affect the positioning of macaques during social interactions. This finding is not consistent with previous research suggesting that dominance rank can affect social laterality in non-human primates [21]. Regarding the sagittal preference, we found no significant correlations between the TI and the dominance hierarchy (CBI). However, for proximity, the *p*-value approached the level of significance considering both the TI (*p* = 0.059) and the ABS-TI (*p* = 0.051), suggesting that higher-ranking macaques are kept more frequently in front than behind. This result supports the hypothesis that keeping conspecifics frontally would facilitate the predictability of intentions and reactions of conspecifics. Indeed, subordinates interacting with a dominant group member have to pay particular attention to its face or body, in order to anticipate aggressive responses [21,22]. Thus, hierarchical rank seems therefore to influence social sagittal positioning but not social laterality in *Barbary macaques*.

## 5. Conclusions

In conclusion, although we reported no biases in the side preference of macaques during social interactions, distinct social behaviors and emotions seem to be associated with a different degree of laterality. For the sagittal preference, the study *Barbary macaques* preferred to keep conspecifics frontally when in proximity, and this preference might be due to the enhanced predictability of the behaviors of other macaques, an improved communication through emotions’ detection as well as to the species social habits. More studies on a greater sample of *Barbary macaques* as well as other non-human primates are needed and should focus also on laterality/sagittal positioning preference during both positive (e.g., affiliative) and negative (e.g., aggressive) social interactions, considering the effect of age, sex and species temperament.

## Figures and Tables

**Table 1:** Side and transversality preference during different social interactions. For each macaque, the table reports name, sex (F = female; M = male) and age (years at the time of data collection). For each social interaction (approach, proximity and affiliative contacts), the table reports the total number of bouts collected to calculate both SI (N_SI_) and TI (N_TI_), the SI (Side Index), the TI (Transversality Index) and the relative *z*-score. Asterisks indicate subjects with a significant laterality preference (right/front: *z*-score ≥ 1.96 or left/rear: *z*-score ≤ −1.96). # Insufficient number of datapoints.

			Approach						Proximity						Affiliative Contacts					
Subject	Sex	Age	N_SI_	SI	*z*-Score	N_TI_	TI	*z*-Score	N_SI_	SI	*z*-Score	N_TI_	TI	*z*-Score	N_SI_	SI	*z*-Score	N_TI_	TI	*z*-Score
Belinda (B)	F	12	44	0.05	0.15	21	−0.52	−2.18 *	49	−0.06	−0.29	20	−0.10	−0.22	21	0.24	0.87	14	−0.57	−1.87
Belle (BL)	F	4	66	−0.24	−1.85	29	0.38	1.86	82	−0.22	−1.88	19	0.26	0.92	42	−0.48	−2.93 *	33	0.27	1.39
Berta (BR)	F	6	47	0.02	0.00	6	0.00	#	53	0.06	0.27	5	0.20	#	31	−0.23	−1.08	2	0.00	#
Buddha (BU)	F	13	17	−0.18	−0.49	18	−0.33	−1.18	16	−0.25	−0.75	9	0.56	#	26	−0.23	−0.98	27	−0.48	−2.31 *
Elly (EL)	F	5	27	−0.11	−0.38	19	0.05	0.00	38	−0.11	−0.49	17	0.18	0.49	36	−0.39	−2.17 *	21	0.05	0
Emma (EM)	F	5	22	0.09	0.21	13	0.08	0.00	20	0.00	0.00	12	0.83	2.6 *	32	0.13	0.53	30	0.20	0.91
Fanny (F)	F	8	54	0.00	0.00	12	0.50	1.44	54	−0.11	−0.68	14	0.00	0.00	15	−0.47	−1.55	2	−1.00	#
Jack (J)	M	4	37	0.30	1.64	26	−0.15	−0.59	31	0.23	1.08	23	0.74	3.34 *	9	0.78	#	17	0.76	2.91 *
Labbraviola (LV)	F	10	35	0.09	0.34	20	0.00	0.00	49	0.06	0.29	15	−0.20	−0.52	10	0.00	0.00	3	−1.00	#
Luke (LAZZ)	M	4	24	−0.25	−1.02	14	0.14	0.27	29	−0.17	−0.74	10	0.60	1.58	5	0.20	#	13	0.23	0.55
Lucky (LK)	M	14	42	0.05	0.15	29	0.38	1.86	47	−0.06	−0.29	16	0.88	3.25 *	25	0.04	0.00	32	−0.13	−0.53
Lucrezia (LU)	F	9	26	−0.38	−1.77	21	0.33	1.31	30	−0.07	−0.18	41	0.17	0.94	22	−0.45	−1.92	35	−0.03	0

## Data Availability

The data presented in this study are available on request from the corresponding author.

## References

[1] MacNeilage P.F., Rogers L.J., Vallortigara G. (2009). Origins of the left & right brain. Sci. Am..

[2] Ghirlanda S., Vallortigara G. (2004). The evolution of brain lateralization: A game-theoretical analysis of population structure. Proc. R. Soc. B Boil. Sci..

[3] Rogers L.J., Vallortigara G., Andrew R.J. (2013). Divided Brains: The Biology and Behaviour of Brain Asymmetries.

[4] Rogers L.J., Zucca P., Vallortigara G. (2004). Advantages of having a lateralized brain. Proc. R. Soc. B Boil. Sci..

[5] Salva O.R., Regolin L., Vallortigara G. (2012). Inversion of contrast polarity abolishes spontaneous preferences for face-like stimuli in newborn chicks. Behav. Brain Res..

[6] Robins A., Lippolis G., Bisazza A., Vallortigara G., Rogers L.J. (1998). Lateralized agonistic responses and hindlimb use in toads. Anim. Behav..

[7] Pellitteri-Rosa D., Gazzola A. (2018). Context-dependent behavioural lateralization in the European pond turtle *Emys orbicularis* (*Testudines, Emydidae*). J. Exp. Biol..

[8] Deckel W.A. (1995). Laterality of aggressive responses in Anolis. J. Exp. Zool..

[9] Hews D.K., Worthington R.A. (2001). Fighting from the right side of the brain: Left visual field preference during aggression in free-ranging male tree lizards (*Urosaurus ornatus*). Brain Behav. Evol..

[10] Nagy M., Ákos Z., Biro D., Vicsek T. (2010). Hierarchical group dynamics in pigeon flocks. Nature.

[11] Karenina K., Giljov A., Ingram J., Rowntree V.J., Malashichev Y. (2017). Lateralization of mother–infant interactions in a diverse range of mammal species. Nat. Ecol. Evol..

[12] Giljov A., Karenina K., Malashichev Y. (2018). Facing each other: Mammal mothers and infants prefer the position favouring right hemisphere processing. Biol. Lett..

[13] Hook M., Rogers L. (1998). Lateralized use of the mouth in production of vocalizations by marmosets. Neuropsychologia.

[14] De Latude M., Demange M., Bec P., Blois-Heulin C. (2008). Visual laterality responses to different emotive stimuli by red-capped mangabeys, Cercocebus torquatus torquatus. Anim. Cogn..

[15] Wallez C., Vauclair J. (2011). Right hemisphere dominance for emotion processing in baboons. Brain Cogn..

[16] Fernández-Carriba S., Loeches A., Morcillo A., Hopkins W.D. (2002). Functional asymmetry of emotions in primates: New findings in chimpanzees. Brain Res. Bull..

[17] Roberts A.I., Murray L., Roberts S.G.B. (2019). Complex sociality of wild chimpanzees can emerge from laterality of manual gestures. Hum. Nat..

[18] Karenina K., Giljov A. (2018). Mother and offspring lateralized social behavior across mammalian species. Prog. Brain Res..

[19] Regaiolli B., Spiezio C., Hopkins W.D. (2018). Asymmetries in mother-infant behaviour in Barbary macaques (*Macaca sylvanus*). PeerJ.

[20] Casperd J.M., Dunbar R. (1996). Asymmetries in the visual processing of emotional cues during agonistic interactions by gelada baboons. Behav. Process..

[21] Baraud I., Buytet B., Bec P., Blois-Heulin C. (2009). Social laterality and ‘transversality’ in two species of mangabeys: Influence of rank and implication for hemispheric specialization. Behav. Brain Res..

[22] Quaresmini C., Forrester G.S., Spiezio C., Vallortigara G. (2014). Social environment elicits lateralized behaviors in gorillas (*Gorilla gorilla gorilla*) and chimpanzees (*Pan troglodytes*). J. Comp. Psychol..

[23] Picaud S., Dalkara D., Marazova K., Goureau O., Roska B., Sahel J.-A. (2019). The primate model for understanding and restoring vision. Proc. Natl. Acad. Sci. USA.

[24] Stidwill D., Optom F.C., Fletcher R., Optom F.C., Orth D. (2010). Normal Binocular Vision: Theory, Investigation and Practical Aspects.

[25] Altmann J. (1974). Observational study of behaviour: Sampling methods. Behaviour.

[26] Thierry B., Bynum E.L., Baker S., Kinnaird M.F., Matsumura S., Muroyama Y., O’Brien T.G., Petit O., Watanabe K. (2000). The social repertoire of Sulawesi macaques. Primate Res..

[27] Deag J.M. (1974). A study of the Social Behaviour and Ecology of the Wild Barbary Macaque, Macaca sylvanus.

[28] Sandri C., Regaiolli B., Vespiniani A., Spiezio C. (2017). New food provision strategy for a colony of Barbary macaques (*Macaca sylvanus*): Effects on social hierarchy?. Integr. Food Nutr. Metab..

[29] Norscia I., Palagi E. (2015). The socio-matrix reloaded: From hierarchy to dominance profile in wild lemurs. PeerJ.

[30] Bang A., Deshpande S., Sumana A., Gadagkar R. (2010). Choosing an appropriate index to construct dominance hierarchies in animal societies: A comparison of three indices. Anim. Behav..

[31] Clutton-Brock T., Albon S., Gibson R., Guinness F. (1979). The logical stag: Adaptive aspects of fighting in red deer (*Cervus elaphus* L.). Anim. Behav..

[32] Michel G.F., Sheu C.-F., Brumley M.R. (2002). Evidence of a right-shift factor affecting infant hand-use preferences from 7 to 11 months of age as revealed by latent class analysis. Dev. Psychobiol..

[33] McGrew W.C., Marchant L.F. (1997). Using the tools at hand: Manual laterality and elementary technology in *Cebus* spp. and *Pan* spp.. Int. J. Primatol..

[34] Boeving E.R., Belnap S.C., Nelson E.L. (2017). Embraces are lateralized in spider monkeys (*Ateles fusciceps rufiventris*). Am. J. Primatol..

[35] Demaree H., Everhart D., Youngstrom E., Harrison D.W. (2005). Brain Lateralization of emotional processing: Historical roots and a future incorporating “dominance”. Behav. Cogn. Neurosci. Rev..

[36] Kuhl J., Kazén M. (2008). Motivation, affect, and hemispheric asymmetry: Power versus affiliation. J. Pers. Soc. Psychol..

[37] Hecht D. (2014). Cerebral lateralization of pro- and anti-social tendencies. Exp. Neurobiol..

[38] Rogers L.J., Vallortigara G. (2015). When and why did brains break symmetry?. Symmetry.

[39] Boccia M. (1989). Comparison of the physical characteristics of grooming in two species of macaques (*Macaca nemestrina* and *M. radiata*). J. Comp. Psychol..

[40] Allanic M., Hayashi M., Furuichi T., Matsuzawa T. (2020). Social influences on grooming site preferences in wild bonobos (*Pan paniscus*) at Wamba, DRC. Primates.

[41] Thierry M., Kaumanns B., Singh W. (2004). Macaque Societies: A Model for the Study of Social Organization.

[42] Thierry B. (2007). Unity in diversity: Lessons from macaque societies. Evol. Anthr. Issues News Rev..

